# Phylogenetic tree construction using trinucleotide usage profile (TUP)

**DOI:** 10.1186/s12859-016-1222-3

**Published:** 2016-10-06

**Authors:** Si Chen, Lih-Yuan Deng, Dale Bowman, Jyh-Jen Horng Shiau, Tit-Yee Wong, Behrouz Madahian, Henry Horng-Shing Lu

**Affiliations:** 1 Key Laboratory of Combinatorial Biosynthesis and Drug Discovery Ministry of Education and School of Pharmaceutical Sciences Wuhan University, Wuhan, China; 2 Department of Mathematical Sciences, University of Memphis, Memphis, TN USA; 3 Institute of Statistics, National Chiao Tung University, Hsinchu, Taiwan; 4 Department of Biological Sciences, University of Memphis, Memphis, TN USA

**Keywords:** Feature frequency profile (FFP), Reading frame, Summary statistics, Phylogenetic tree construction, Tree comparison

## Abstract

**Background:**

It has been a challenging task to build a genome-wide phylogenetic tree for a large group of species containing a large number of genes with long nucleotides sequences. The most popular method, called feature frequency profile (FFP-*k*), finds the frequency distribution for all words of certain length *k* over the whole genome sequence using (overlapping) windows of the same length. For a satisfactory result, the recommended word length (*k*) ranges from 6 to 15 and it may not be a multiple of 3 (codon length). The total number of possible words needed for FFP-*k* can range from 4^6^=4096 to 4^15^.

**Results:**

We propose a simple improvement over the popular FFP method using only a typical word length of 3. A new method, called Trinucleotide Usage Profile (TUP), is proposed based only on the (relative) frequency distribution using *non-overlapping* windows of length 3. The total number of possible words needed for TUP is 4^3^=64, which is much less than the total count for the recommended optimal “resolution” for FFP. To build a phylogenetic tree, we propose first representing each of the species by a TUP vector and then using an appropriate distance measure between pairs of the TUP vectors for the tree construction. In particular, we propose summarizing a DNA sequence by a matrix of three rows corresponding to three reading frames, recording the frequency distribution of the non-overlapping words of length 3 in each of the reading frame. We also provide a numerical measure for comparing trees constructed with various methods.

**Conclusions:**

Compared to the FFP method, our empirical study showed that the proposed TUP method is more capable of building phylogenetic trees with a stronger biological support. We further provide some justifications on this from the information theory viewpoint. Unlike the FFP method, the TUP method takes the advantage that the starting of the first reading frame is (usually) known. Without this information, the FFP method could only rely on the frequency distribution of overlapping words, which is the average (or mixture) of the frequency distributions of three possible reading frames. Consequently, we show (from the entropy viewpoint) that the FFP procedure could dilute important gene information and therefore provides less accurate classification.

## Introduction

The construction of phylogenetic trees, based on the whole-genome information, is one of the challenging problems in computational biology. The difficulty is how to best utilize genome-wide DNA information. Each species has many genes and each gene can have a long DNA sequence. To capture the essential whole-genome DNA information, many different methods have been proposed. To quantify the closeness between two species, one can consider various distance functions to measure the closeness between two DNA sequences. We review some popular methods as follows.

Traditional methods were based on the classical sequence alignment methodology; see, for example, [1]. For each potential alignment, a score of similairity/dissimilarity is assigned to each base pair and an alignment score of the two sequences is obtained by summing the scores across all pairs in the sequences. The alignment with the highest score is outputted as the final aligning result. The evolutionary distance measure between two organisms is the similarity/dissimilarity of their proteinic or genomic/genic sequences. In general, such alignment-based methods would have a huge computational cost and are infeasible for entire proteomic/genomic sequence comparison. One common practice is using some selected gene(s) to represent the whole genome information. However, there is typically no general agreement about the choice of one or multiple representative genes. Additionally and most importantly, it can be hard to find common genes in all organisms under study, especially when the organisms are phylogenetically distant from one another.

To overcome the difficulties of the alignment-based methods, various alignment-free methods for phylogenetic tree construction have been proposed in the literature. One popular method is word-based, which involves counting the frequency of words of a specific length in the whole genome DNA sequence. See, for example, [2–4, 6, 7]. Most of the word-based research works have been focused on two directions: (i) choice of an optimal word size [4–6, 8] and/or (ii) choice of a proper distance measure between two word frequency distributions [2, 3, 9–11]. As pointed out in [4], some of these methods were variations of known techniques for comparing two text strings, also known as Latent Semantic Analysis (LSA). LSA is a popular technique in natural language processing used to analyze the similarity/dissimilarity between a set of documents [12]. In [4], a feature frequency profile (FFP) of length *k*, denoted by FFP-*k*, was obtained by scanning the DNA sequence with overlapping windows of size *k* to find the *k*-tuple frequency distribution (with 4^*k*^ possible values) over the DNA sequence. [4] proposed estimating the optimal length or resolution of the features by using the delimiter-stripped text from some popular English books. They then used Jensen-Shannon Divergence measure as a distance between two FFPs. There are several obvious problems with this approach: (i) The optimal length could depend on the character strings considered and there is a wide range of possible lengths, say, between 6 to 15. (ii) The obtained optimal length has little, if any, biological support. (iii) If the optimal word size is large, the vector size of the corresponding FFP would grow exponentially.

For a DNA sequence, the most natural (and biologically sensible) word length is 3, which is clearly outside the optimal range of 6 to 15 for the word length as found in [4]. Denote the feature frequency profile for words of length 3 by FFP-3. The FFP-3 (or other word lengths) for a DNA sequence may fail to retain its essential information about the higher order (dimensional) structure between successive nucleotides. Keeping the word length at 3, we propose a simple modification on the counting of the word frequencies for trinucleotides (word of 3 nucleotides). The basic idea of our approach is to record the separate information from three reading frames (RFs), where the second and the third RFs are constructed from the first (original) RF by shifting one and two nucleotides, respectively. Strictly speaking, the word “codon” is generally restricted to the description of the trinucleotides on the first reading frame. In this paper, we will use the term “translation-triplet”, or simply TT, to denote either the codon in the first reading frame, or the trinucleotide in the second and third reading frames. Specifically, the proposed summary statistic is a matrix of three vectors of size 64(= 4^3^) each: the first vector is the frequency distribution of the codons (of length 3, *non-overlapping*) corresponding to the first reading frame; the second and third vectors are constructed similarly from the corresponding second and third reading frames, respectively.

The rest of the paper is organized as follows. First, we describe the data under study, including the data source and format. In total, there are 56 species in this study. These species have potentially different numbers of genes and the genes have a large variation in length. Next, we discuss the general framework for alignment-free tree construction methods. We propose a summary measure function that retains the vital information associated with each species. We show in our study later that this summary measure function, called the vector-extracting function, yields a matrix based on three reading frames that can retain key information even with additional data reduction. While several methods have been proposed by researchers [13–15], they are not as intuitive as ours and often are computationally time-consuming. We also propose a simple and heuristic numerical measure for making a formal comparison among various trees. Finally, various vector-extracting functions are shown to yield consistent phylogenetic construction whereas the popular FFP-3 vector does not yield a tree that is consistent with other known species classifications. Using the trees constructed, we show the usefulness of our proposed distance measure between trees.

## Description of data

### Species included in the study

In this paper, we select a broad range of bacteria from several well-studied clones of eight different genera from three distinct subphyla of the Proteobacteria. To prevent bias due to variations of individual genomes, multiple genomes from different strains of a species were selected. The genera Orientia (1 species), Rickettsia (9 species/strains), and Wolbachia (2 strains) are members of a monophyletic class ([16]). These bacteria were used to represent the *α*-Proteobacteria subphylum. The 5 species/strains from the monophyletic genus Neisseriae [17] were used to represent the *β*-Proteobacteria subphylum. The monophyletic family of Escherichia (22 species/strains), Shigella (4 species), Salmonella (4 strains), and a separate monophyletic genus of Yersinia (9 species/stains) were selected to represent the *γ*- Proteobacteria. It should be noted that the Escherichia and Shigella are now considered as the same genus [18]. Escherichia and Salmonella are diverse from each other about 150 million years ago [19]. Most experts agree that the *β*- and *γ*-Proteobacteria are more closely related to each other than the *α*-Proteobacteria [20]. In total, 56 species are selected.

### Source of data and processing methods

The FASTA.ffn files of 56 bacterial genomes were downloaded from the Comprehensive Microbial Research website (lbrinkac@jcvi.org). Each data file is in FASTA format and it contains the coding sequences for mRNAs in the genome, excluding the regulatory sequences and the sequences for tRNA and rRNA. Each data file has a various number of segments (or genes), depending on the genome size. In this paper, we use “segment” and “gene” interchangeably because each segment represents the coding sequence for a gene. A segment has two parts in its structure. The first part is a text paragraph describing the information about the gene such as name, location in chromosome, etc. The second part is a letter sequence of “A”, “T”, “C”, and “G”, which is the nucleotide sequence in the DNA strand. The following example is a gene segment from E coli K12 DH10B:



One can extract the nucleotide sequence from the data file using a downloadable *R* package “*seqinr*” with its function “*read.fasta()*”. We perform additional post-processing procedures on the nucleotide sequence as described next.

The genetic code of 64 codons, represented by three nucleotides, is reduced to 20 distinct amino acids, which are the functional building blocks of proteins. Some small percentage (less than one percent) of nucleotide sequences extracted from the data was excluded as non-informative. The gene count and a gene length summary (including minimum, average, and maximum) for each of the 56 bacterial species are listed in Table 1.

**Table 1:** Gene count and the minimum, average, and maximum of gene lengths for each of 56 species

Strain (Species)	Gene	Min	Mean	Max
	Count			
Escherichia_coli_O15_7_H7_VT2Sakai	5361	45	903.5	15876
Escherichia_coli_0127_H6_E2348_69	4703	45	929.7	9672
Escherichia_coli_536	4685	66	934.7	9729
Escherichia_coli_55989	4919	45	929.4	9492
Escherichia_coli_BL21_DE3	4319	36	937.5	7104
Escherichia_coli_BW2952	4084	45	954.8	7077
Escherichia_coli_B_REL606	4209	45	953.7	7152
Escherichia_coli_C_ATCC_8739	4200	75	974.7	6342
Escherichia_coli_E24377A	4755	90	907.1	6891
Escherichia_coli_ED1a	5123	45	900.6	9492
Escherichia_coli_IAI1	4443	45	942.0	6444
Escherichia_coli_IAI39	4892	45	931.1	9492
Escherichia_coli_K_12_substr_DH10B	4200	45	945.6	7104
Escherichia_coli_K_12_substr_MG1655	4321	45	946.5	7077
Escherichia_coli_K_12_substr_W3110	4337	45	950.7	8622
Escherichia_coli_O157_H7_EC4115	5315	93	873.0	7863
Escherichia_coli_S88	4847	45	924.0	9492
Escherichia_coli_SE11	4679	45	929.2	5421
Escherichia_coli_SMS_3_5	4743	75	935.4	8802
Escherichia_coli_UMN026	4907	45	942.9	20778
Escherichia_coli_UTI89	5066	66	911.3	9789
Escherichia_fergusonii_ATCC_35469	4319	45	954.2	21669
Neisseria_gonorrhoeae_FA_1090	2002	111	845.4	5934
Neisseria_meningitidis_053442	2020	93	853.9	5364
Neisseria_meningitidis_FAM18	1975	87	916.5	6090
Neisseria_meningitidis_MC58	2063	69	871.9	8112
Neisseria_meningitidis_Z2491	1993	93	900.1	6048
Orientia_tsutsugamushi_Boryong	2179	30	796.1	6900
Rickettsia_conorii_Malish_7	1374	126	746.4	6066
Rickettsia_prowazekii_Madrid_E	834	126	1006.9	7023
Rickettsia_akari_Hartford	1259	63	741.9	5682
Rickettsia_bellii_OSU_85-389	1476	78	831.9	4752
Rickettsia_bellii_RML369-C	1429	123	907.8	5946
Rickettsia_felis_URRWXCal2	1400	123	889.4	9369
Rickettsia_rickettsii_Iowa	1384	54	701.7	5622
Rickettsia_rickettsii_Sheila_Smith	1345	63	713.4	6750
Rickettsia_typhi_wilmington	838	75	1002.1	6996
Salmonella_enterica_serovar_Typhi_CT18	4395	42	910.1	10875
Salmonella_typhimurium_LT2_SGSC1412	4451	45	947.6	16680
Salmonella_enterica_Choleraesuis	4445	66	898.3	16680
Salmonella_enterica_Paratypi_ATCC_9150	4093	66	924.8	13683
Shigella_boydii_Sb227	4142	45	880.2	4962
Shigella_dysenteriae	4277	45	789.9	4767
Shigella_flexneri_2a_301	4436	42	912.4	5673
Shigella_sonnei_Ss046	4224	45	919.9	4962
Wolbachia_pipientis_wMel	1271	93	857.0	8532
Wolbachia_pipientis_wBm	805	129	899.4	8520
Yersinia_enterocolitica_8081	4060	84	962.1	9486
Yersinia_pestis_Angola	3837	114	902.1	9492
Yersinia_pestis_Antiqua	4167	69	949.0	11118
Yersinia_pestis_biovar_Medievalis_91001	3895	63	962.3	11133
Yersinia_pestis_CO92	4008	45	973.0	11118
Yersinia_pestis_KIM_10	4090	45	937.8	11133
Yersinia_pestis_Pestoides_F	3850	87	962.9	13971
Yersinia_pseudotuberculosis_IP32953	3974	45	998.5	16872
Yersinia_pseudotuberculosis_IP_31758	4124	48	952.2	14862

## Phylogenetic tree construction methods

### Alignment-free tree construction

We let *S*_*i*_ denote the *i*-th strain in the study and use the notation *S*_*i*_∼*S*_*j*_ to denote that the strains *S*_*i*_ and *S*_*j*_ are closely related to each other. To measure the closeness of two strains *S*_*i*_ and *S*_*j*_, we first find a summary function *f*() to produce a general summary measure for each strain *S*_*i*_:

$$\mathbf{M}_{i}= f(S_{i}) $$

and then find a distance function *d*() satisfying the following condition:

$$S_{i} \sim S_{j} \Leftrightarrow d(\mathbf{M}_{i}, \mathbf{M}_{j}) \approx 0. $$

That is, if two strains, *S*_*i*_ and *S*_*j*_, are closely related to each other, then their summary measures, **M**_*i*_=*f*(*S*_*i*_) and **M**_*j*_=*f*(*S*_*j*_), are expected to be close to each other as well.

The success (or failure) of the tree construction depends heavily on the choice of an appropriate summary function, *f*(), to represent and characterize the long whole-genome DNA sequence of the species. Generally speaking, there is a trade-off between the compactness and completeness of the chosen summary function. Clearly, the most complete statistic is the whole-genome DNA sequence itself, but it is too big to be practical for a meaningful genome-wide comparison between two species. On the other hand, choosing a simple summary function may fail to retain the vital information for a proper comparison or tree construction. We will consider some possible summary functions later.

If the summary measure **M**_*i*_ is a vector, then we can choose *d*() to be any distance function. For example, the usual Euclidean distance

$$d({x},{y})= \left(\sum_{i=1}^{n}(x_{i}-y_{i})^{2} \right)^{1/2} $$

or the city block distance (Manhattan distance)

$$d({x}, {y})= \sum_{i=1}^{n} |x_{i}-y_{i}|, $$

where *x*=(*x*_1_,*x*_2_,…,*x*_*n*_) and *y*=(*y*_1_,*y*_2_,…,*y*_*n*_). In our experience, there is not much difference between these two choices of the distance measure. In this paper, we choose the city block distance (Manhattan distance).

In our proposed method, there is a slight complication for phylogenetic tree construction—our proposed summary measure **M**_*i*_ is a matrix instead of a vector. There is no standard way to define the distance between two matrices. One possible solution is to extract rows and/or columns from the summary matrix and convert them into a vector. Denote this vector extracting function by *v*(). Then, given two summary matrices, **M**_*i*_ and **M**_*j*_, we can define the distance between them by *d*(*v*(**M**_*i*_),(*v* **M**_*j*_)). Several reasonable choices of the vector extracting function *v*() will be discussed later.

For a proper choice of the summary function *f*(), vector extracting function *v*(), and distance function *d*(), one would expect

$$S_{i} \sim S_{j} \Leftrightarrow d\left(v\left(\mathbf{M}_{i}\right), v\left(\mathbf{M}_{j}\right)\right) \approx 0. $$

Having chosen these functions, we then perform hierarchical clustering with complete linkage. An open source software “*Cluster 3.0*” developed by Michael Eisen from Stanford University was used to generate the clustering results. In addition, we use GNU GPL v2 software “*Java TreeView 1.1.6r2*” by Alok Saldanha to display the hierarchical dendrograms. Both programs can be downloaded at http://bonsai.hgc.jp/~mdehoon/software/cluster/software.htm.

In the following, we first discuss the proposed choice of the summary function *f*() and then we consider various choices of the vector extracting function *v*().

### Trinucleotide usage profile (TUP)

Given a gene with a sequence of nucleotides (“A”, “C”, “G”, “T”), there are several reasonable ways to summarize the nucleotide sequence. For example, we can group the nucleotides in the sequence in *non-overlapping* triplets and then count the frequency for each of the 64 possible triplets. Another popular summary measure is the frequencies of the 64 triplets in the set of the *successive overlapping* triplets of the sequence. The latter is a special case of the aforementioned feature frequency profile FFP-*k* with *k*=3. The vector of 4^*k*^ frequency counts is commonly referred to as the FFP-*k* vector [4]. As mentioned earlier, the recommended word length *k* for the FFP-*k* vector is in the range of 6 to 15 depending on the sequence under study [4]. For *k* = 3, a natural codon length, the obtained FFP-3 vector may fail to retain vital information contained in the whole DNA sequence, as evidenced later with an example as well as by information theory.

In this paper, we propose a simple but essential modification on the FFP-3 method. For each strain (species), we find the frequency distribution of 64 TTs in each of the three reading frames and create a summary matrix of 3 rows and 64 columns as follows.

For each gene of a strain, we count the frequencies of the 64 TTs (non-overlapping) in each of its three reading frames to create a *genic* 3 × 64 TT count matrix. A *genomic* (genome-wide) TT count matrix of a species is simply the sum of all its genic TT count matrices. Specifically, let *G*_*i*_ denote the number of genes in the *i*-th genome and **c**_*ig*_ denote the genic TT count matrix of the *g*-th gene in the *i*-th genome for *g*=1,2,…,*G*_*i*_ and *i*=1,2,…,56. Summing over all genes, we have $\mathbf {C}_{i} = \sum _{g=1}^{G_{i}} \mathbf {c}_{ig}$ as the TT count matrix of the *i*-th genome.

For strain *S*_*i*_, we scale its count matrix **C**_*i*_ by dividing each row element by the corresponding row total and denote the normalized matrix (of size 3x64) by **M**_*i*_. Let *T*_*i*_ be the total TT counts of the first row of **C**_*i*_. Then the total row counts of the second and the third rows of **C**_*i*_ are *T*_*i*_−1 when we omit the nucleotides that can not be in triplet due to frame shift in the second and third reading frame of a gene segment. To illustrate this, we take the aforementioned gene (E coli K12 DH10B) as an example. In the first reading frame, all the nucleotide triplets are “GTG”, “AAA”, “AAG”, …, “TAA”. However, when we shift the frame one nucleotide to the right to get the second reading frame, the triplet sequence starts with “TGA” and ends with “GCT”. So the first nucleotide (“G”) and the last two nucleotides (“AA”) can not be in triplet. These three nucleotides are excluded from the calculation. Similarly, in the third reading frame, the first two nucleotides (“GT”) and the last nucleotide (“A”) are omitted. Therefore, the total TT count for the first reading frame is one more than that for the second or the third reading frame. In practice, *T*_*i*_ is a very large number, hence we can obtain the normalized matrix simply by

$$\mathbf{M}_{i} = \mathbf{C}_{i}/T_{i}. $$

For the remainder of this paper, we refer to the summary matrix **M**_*i*_ as the Trinucleotide Usage Profile (TUP) matrix.

### Vector extracting functions

We now let a strain/bacterium be represented by a TUP matrix of size 3x64 containing the genome-wide proportions of all the 64 types of TTs corresponding to the three reading frames. To find the distance between two TUP matrices, **M**_*i*_ and **M**_*j*_, we need to choose a proper vector extracting function, *v*(), and compute *d*(*v*(**M**_*i*_),*v*(**M**_*j*_)). The following are some examples.

1. Extract any of the three rows from the TUP matrix. The vectors corresponding to the first, second, and third RFs are designated as the TUP-R1 vector, TUP-R2 vector, and TUP-R3 vector, respectively.
2. Extract all of the three rows from the summary matrix and concatenate them into a vector of 192 elements. The value of each element is the proportion of the combined TTs from the three RFs (3x64) of that bacterium. This vector is designated as the TUP-All vector.
3. Extract the columns from the TUP matrix corresponding to a specific amino acid or stop codons. For example, we can extract the three columns from the summary matrix corresponding to the three stop codons (“TAA”, “TAG”, and “TGA”) and convert them into a vector of 9 elements. This approach was used successfully in [21] for a phylogenetic tree construction. It is interesting to observe that extracting columns corresponding to any specific amino acid, in general, has slightly inferior phylogenetic tree construction than those using stop codons. According to [21], the stop codons serve a vital role in gene expression and avoidance of transcriptional mistakes and it could offer a shortcut for whole genome analysis.
4. Choose the output vector to be the sum of the three rows in the TUP matrix. This in fact gives the FFP-3 vector in [4] (see also [22]). Recall that the FFP-3 vector counts the occurrences of each of the 64 TTs by scanning the reading frame with moving window of three nucleotides to form a count vector of length 64. Therefore, this count vector is mathematically equivalent to the sum of 3 rows of our 3x64 TT count matrix. So the FFP-3 method can be viewed as performing a vector extracting function on the TUP matrix. However, the study showed (later) that the tree formed by the FFP-3 method yields a biologically inconsistent phylogenetic tree.

While choosing a simpler vector extracting function can provide more compact statistics, it may not retain or characterize certain key information contained in the summary matrix (and the original sequence). Consequently, the constructed phylogenetic tree may not be close to those trees with stronger biological support.

## Results and discussion

Four phylogenic trees were constructed using vectors with (1) TUP-R1 (2), TUP-R2, (3) TUP-R3, and (4) TUP-All, respectively. Hierarchical correlation (city block, complete linkage) was used for clustering.

### Constructed trees using various TUP vectors

**Fig. 1:**
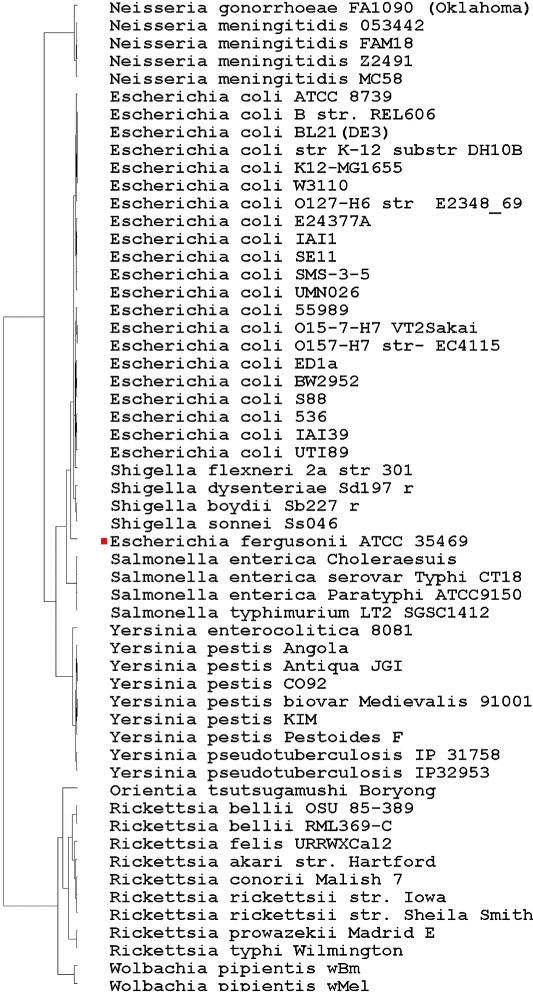
Phylogenic tree based on the TUP-R1 vector

**Fig. 2:**
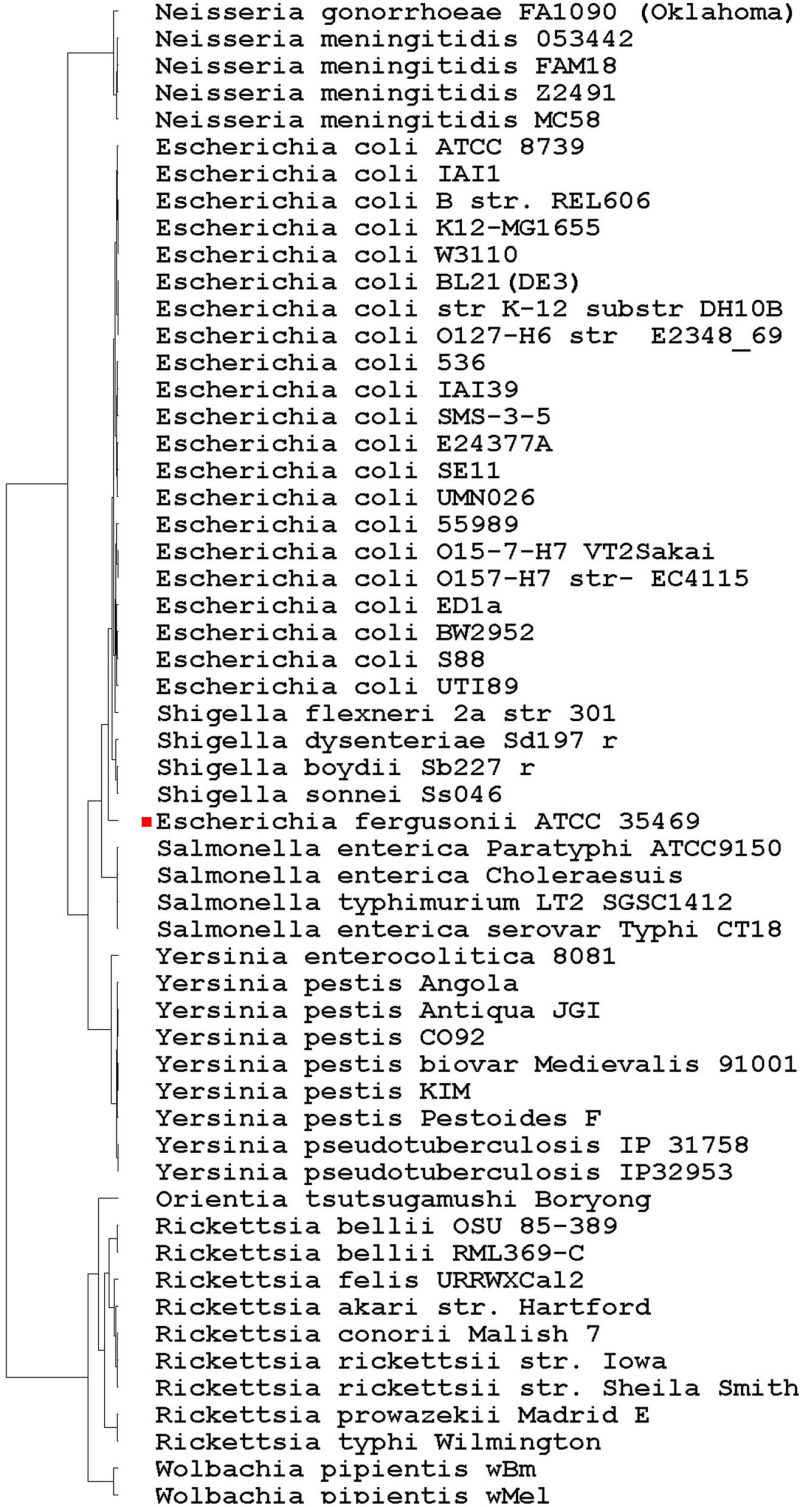
Phylogenic tree based on the TUP-R2 vector

**Fig. 3:**
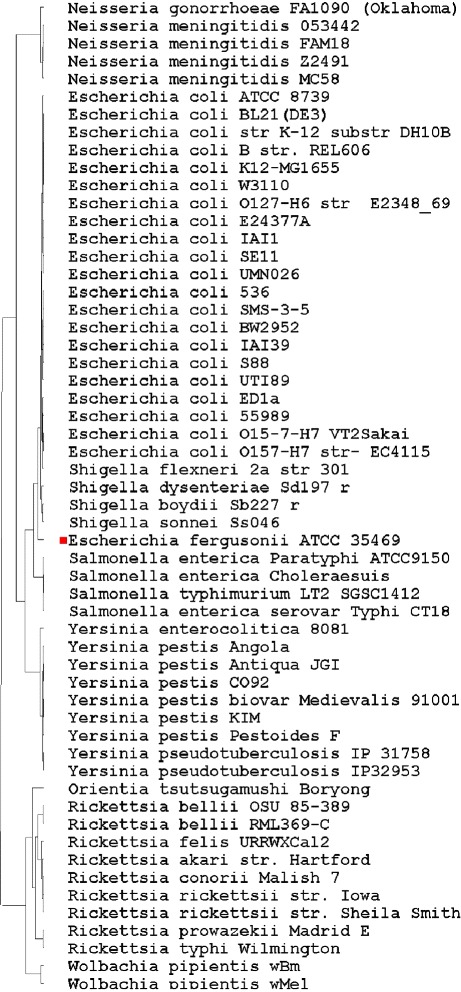
Phylogenic tree based on the TUP-R3 vector

**Fig. 4:**
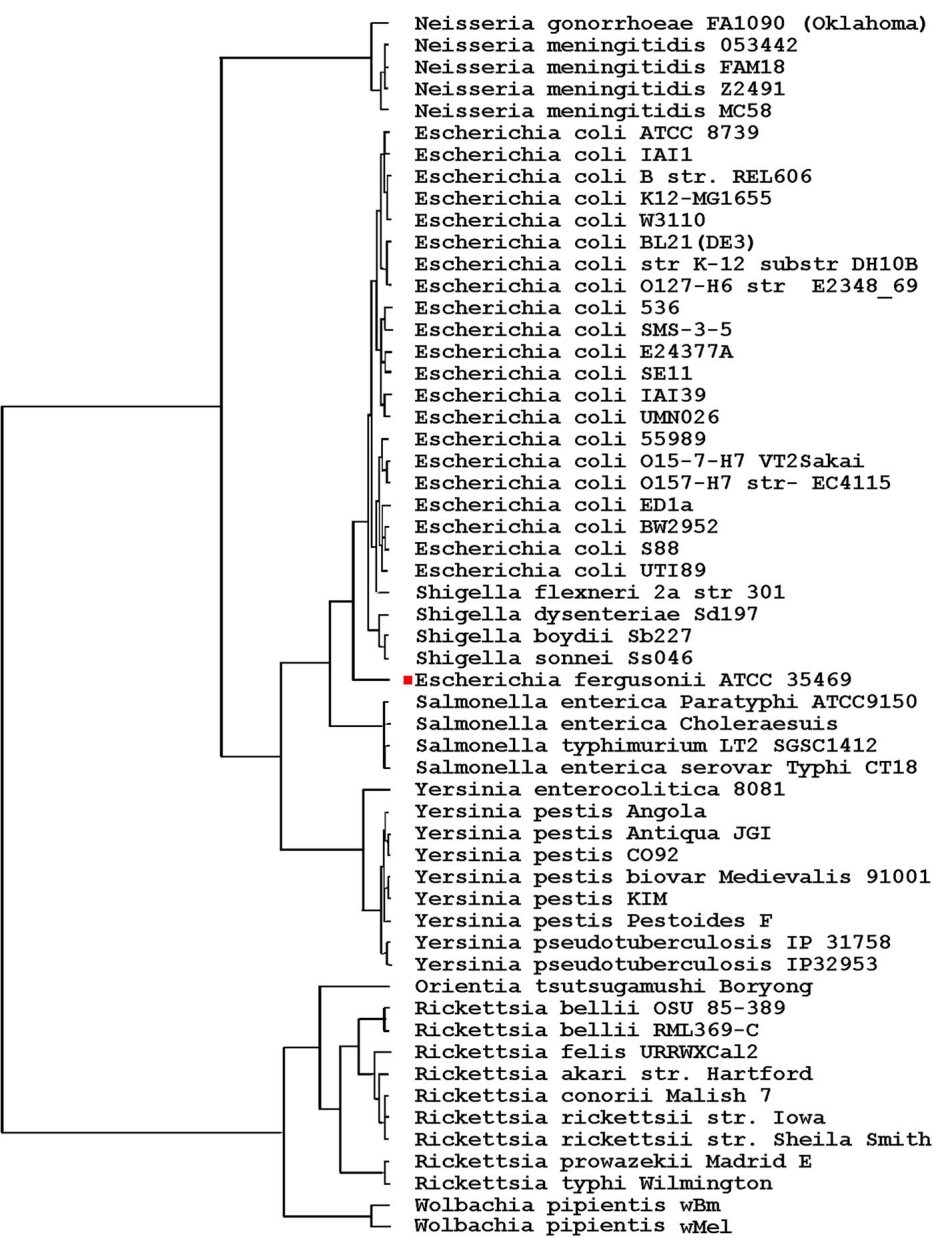
Phylogenic tree based on TUP-All vector

The phylogenetic trees constructed using the four forms of vector extracting functions are shown in Figs. 1, 2, 3 and 4 respectively.

All four trees show consistent and similar patterns. The lab strain E. coli K12-MG1655 and its clones BL21(DE3), W3110, and K12 (DH10B) are always grouped together. However, some wild-type strains, such as the Enterophathogeic strains O127-H6 and the commensal IAI1 strains, are also found to be closely associated with these lab-strains. This finding should not be surprising as the genes of most escherichial strains were the result of lifestyle adaptations [27]. Despite the genome reduction of these lab-strains, their overall genomic vectors might still be comparable to their wild-type strains. The four trees are all in accordance with current knowledge of evolution from the species taxa level. Before giving additional biological interpretations, we first explain why the phylogenetic signals in the vectors TUP-R1, TUP-R2, TUP-R3, and TUP-All are strong, despite the great variation in their numerical values.

The TUP-R1 vector is the distribution of the 64 *non-overlapping* codons, starting at its first reading frame of each gene, on the genome-wide DNA sequence. While TUP-R1 is a reasonable summary statistic for the DNA sequence, it cannot detect TT permutations because TT permutations do not change the distribution of the 64 codons. Likewise, the vectors of TUP-R2 and TUP-R3 are the distributions of the 64 TTs obtained from scanning the second and the third RFs, respectively. Note that the resulting count vectors are quite different due to the shift. Because the three RFs are essentially the *same* (long) DNA sequences, we would expect similar trees to be drawn even with three quite different vectors. On the other hand, the TUP-All vector contains more complete information and it can even detect TT permutations in the whole genome DNA sequence.

### Biological interpretation of the constructed trees

As mentioned earlier, all four tress (Figs. 1, 2, 3 and 4 based on TUP-R1, TUP-R2, TUP-R3, and TUP-All, respectively) constructed are very similar to each other. Therefore, for biological interpretations of the constructed trees, we only discuss in the following the tree constructed by the TUP-R1 vectors as shown in Fig. 1. This tree correctively organizes the bacteria from the three subphyla according to their natural histories. Among the *γ*-Proteobacteria, all the Escherichia/Shigella species are grouped into one tight clade, which is in perfect agreement with the current views on these two genera [18]. E. fergusonii is the most remote member of this clade. The 4 strains of Salmonella are grouped into one tight clade and are closely associated with Escherichia. The correlation between the Escherichia/Shigella group and the Salmonella group is in line with the current view of their natural classification [19]. The 9 species of Yersinia form a tight group, with Y. enterocolitica being the most remote member of this group. This Yersinia clade is distinctly separated from the Escherichia/Salmonella group.

The 5 species of the Neisseriae are members of the *β*-Proteobacteria. They form a distinct branch but are more closely related to the *γ*-Proteobacteria. Although N. gonorrhoeae and N. meningitidis are often difficult to distinguish [23], the codon distributions of these two species are clearly distinguishable.

Within the *α*-Proteobacteria branch, all the Rickettsia species are grouped together. The placing of the Orientia as an extended family of the Rickettsia is in perfect agreement with the literature [24]. The placing of the two parasitic Wolbachia near the Rickettsia/Orientia branch is also in good agreement with the current phylogenetic assignment of this group of bacteria [16, 25, 26].

### Comparison with the FFP-3 method

**Fig. 5:**
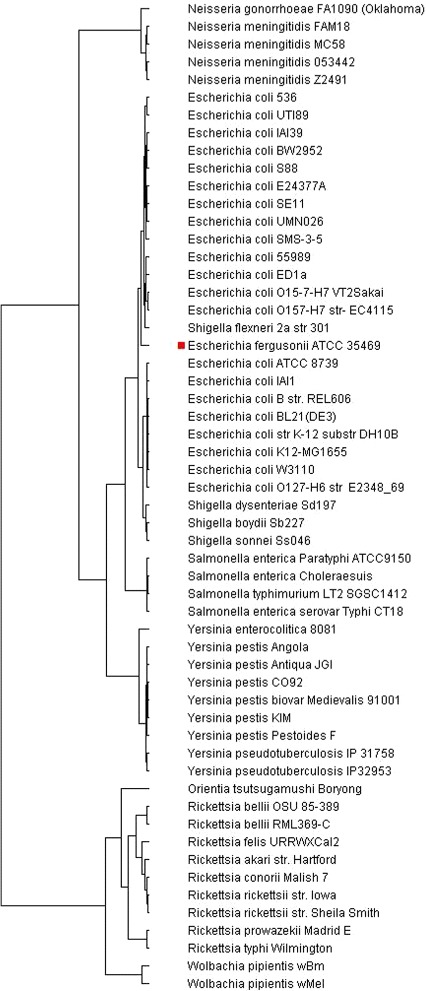
Phylogenic tree based on the FFP method with length 3

For the purpose of comparison, we also perform the grouping of bacteria based on the FFP-3 vector, a special case in [4]. Figure 5 is the tree constructed by the 56 FFP-3 vectors. This tree shows that the phylogenetic signals in the genome are much weaker than the phylogenetic signals in the protein-coding genes. Although the three subphyla could be distinguished by the nucleotide-triples ratios, their resolutions in separating bacterial groups are poor. Furthermore, it could not separate organisms at the lower taxa. For example, the Shigella strains are less similar to the Escherichia strains.

Unlike Figs. 1, 2, 3 and 4, Fig. 5 has the strain “E fergusonii ATCC 35469” (marked with a red dot) *wrongly* clustered within “E coli strains” in the constructed tree. As the FFP-3 vector is (essentially) the sum of three TUP vectors, it may dilute “key information” in DNA sequences. Thus it is very likely that the cause of the mis-classification could be attributed to the vector extracting function used in constructing the tree.

On the other hand, a statistic (e.g., TUP-R1, TUP-R2, TUP-R3, TUP-ALL, or FFP-3 vector) is more effective in classification if it is “less random” across the genes within the same species. Entropy is a popular measure for the randomness, hence it is suitable for comparing the performance of various classification variables. Next, we show theoretically and empirically that the FFP-3 method indeed has a higher entropy (more random) than all the TUP methods.

### Comparing entropy among various methods

Let *X* be a random variable taking *m* possible values, *t*_1_,*t*_2_,…,*t*_*m*_, with *P*(*X*=*t*_*i*_)=*p*_*i*_ for *i*=1,…,*m*. In this paper, *m*=64 and *X* represents the summary vector using TUP or FFP procedure.

The entropy associated with probability vector ***p***=(*p*_1_,*p*_2_,…,*p*_*m*_)$\left (\sum _{i=1}^{m} p_{i}=1\right)$ is

$$H(\boldsymbol{p}) = - \sum_{i=1}^{m} p_{i} \log(p_{i}). $$

It is straightforward to show that $\left (- \frac {\partial ^{2} H}{\partial p_{i} \partial p_{j}} \right)$ is a positive definite matrix, implying that *H*(***p***) is a concave function of ***p***. Consequently, for any two probability vectors ***p*** and ***q*** and for 0<*w*<1, we have

$$H(w\boldsymbol{p} + (1-w) \boldsymbol{q}) \ge wH(\boldsymbol{p}) + (1-w)H(\boldsymbol{q}) $$

for the mixture distribution of *X* (with probability vector ***p***) and *Y* (with probability vector ***q***) given by *Z* *X*+(1−*Z*)*Y* with *P*(*Z*=1)=*w*=1−*P*(*Z*=0).

Note that FFP-3 can be considered as the mixture distribution with equal weights of TUP-R1, TUP-R2, and TUP-R3. Based on this characterization, we have the following observations.

1. The sample entropies calculated for TUP-R1, TUP-R2, and TUP-R3 are of similar magnitudes, which may explain their similar classification power and similar constructed trees.
2. Since FFP-3 is the mixture distribution with equal weights of TUP-R1, TUP-R2, and TUP-R3, the entropy for FFP-3 is larger than the average entropy of the three TUPs. Thus FFP-3 has a higher entropy than at least one of the TUPs. Since all three TUPs have similar entropies, FFP-3 is expected to have a higher entropy than all of them. As mentioned earlier, using a more “random” statistic to represent a species is less likely to be a good characterization/classification of the given species. This may help to explain why the tree constructed by FFP-3 has less biological support than the tree constructed by using TUP-R1, TUP-R2, or TUP-R3.
3. For the purpose of illustration, we consider two examples below. The first one is a real data example and the second one is a simple artificial example with an extreme case.
  - For the E coli K12 DH10B example shown earlier, the entropy for three reading frames, R1, R2, and R3, are 3.750678, 3.71317, and 3.859847, respectively. The entropy for FFP-3 is 3.995315, which is larger than the entropies of all three reading frames.
  - For an artificial example, we consider the DNA sequence of “ACTACTACTACTACTACTACT...”. The TUP-R1 will produce a probability vector with probability 1 concentrating at “ACT” and hence the entropy is 0. Similarly, TUP-R2 and TUP-R3 also have zero entropy with concentration values at “CTA” and “TAC”, respectively. On the other hand, FFP-3 will produce a probability vector with probability 1/3 concentrating at each of three possible values, “ACT”, “CTA”, and “TAC”; hence the entropy is log(3), obviously larger than the zero entropy of the three TUPs.

### Proposed method for measuring “closeness between trees”

When the number of strains under study is large, it could be tedious to “visualize” the closeness of many variously constructed phylogenetic trees. We propose a numeric measure for the closeness between two trees. Let **M**_*i*_ be the TUP matrix for strain *S*_*i*_,*d*() be the distance function, and *v*() be the vector extracting function for the construction of the phylogenetic tree. Define a large vector (of size ${56 \choose 2}=1540$) of pairwise distances between any two strains, say, *S*_*i*_ and *S*_*j*_, as

$$\mathbf{T}({v}) = \left[d(v(\mathbf{M}_{i}), v(\mathbf{M}_{j})), 1 \leq i < j \leq 56 \right]. $$

For two different vector extracting functions, say, *v*_1_() and *v*_2_(), we can compute two vectors $\mathbf {T}(_{v_{1}})$ and $\mathbf {T}(_{v_{2}})$. If the resulting phylogenetic trees are similar to each other, the “distance” (again, in Euclidean distance or city block distance) between $\mathbf {T}(_{v_{1}})$ and $\mathbf {T}(_{v_{2}})$, $d(\mathbf {T}(_{v_{1}}), \mathbf {T}(_{v_{2}}))$, should be small (and vice versa). Next, we use this proposed measure to compute the distance between each pair of the trees constructed.

### Numeric results for “closeness between trees”

To evaluate the “closeness” among the trees, we use the current study as an example. Let *v*_*all*_,*v*_1_,*v*_2_,*v*_3_, and *v*_FFP-3_ be the vector extracting functions corresponding to TUP-All, TUP-R1, TUP-R3, TUP-R3, and FFP-3, respectively. Table 2 summarizes all the pairwise distances among the five trees constructed.

**Table 2:** Pairwise distances among various trees

	$\mathbf {T}(_{v_{1}})$	$\mathbf {T}(_{v_{2}})$	$\mathbf {T}(_{v_{3}})$	$\mathbf {T}(_{v_{\text {FFP-3}}})$
$\mathbf {T}(_{v_{all}})$	12.08	7.97	11.89	108.36
$\mathbf {T}(_{v_{1}})$		16.17	22.90	117.82
$\mathbf {T}(_{v_{2}})$			14.43	110.49
$\mathbf {T}(_{v_{3}})$				96.74

The distances $d(\mathbf {T}(_{v_{all}}), \mathbf {T}(_{v_{i}}))$ for *i*=1,2,3 are 12.08,7.97,11.89, respectively, which are much smaller than the distance $d(\mathbf {T}(_{v(_{all}})), \mathbf {T}(_{v_{\text {FFP-3}}})) (=108.36)$. The distances between $\mathbf {T}(_{v_{\text {FFP-3}}})$ and the other three trees, $\mathbf {T}(_{v_{1}})$, $\mathbf {T}(_{v_{2}})$, and $\mathbf {T}(_{v_{3}})$, are 117.82,110.49, and 96.74, respectively, which are also large. This is consistent with previous observation that the tree in Fig. 5, constructed using $\mathbf {T}(_{v_{\text {FFP-3}}})$, is far different from the trees in Figs. 1, 2, 3 and 4, which have a stronger biological support.

## Summary and extension

In this paper, we proposed a new alignment-free method for constructing a phylogenetic tree based only on the TUPs, the Trinucleotides Usage Profiles, of the genome-wide DNA sequences under study; and each TUP vector represents the (relative) frequency distribution of the 64 trinucleotides obtained by scanning over each of the DNA sequences using non-overlapping windows of length 3. Clearly, the TUP method is slightly more efficient computationally than the popular feature frequency profile FFP-*k* method with *k*=3 because the latter counts the frequency distribution for the overlapping windows of the same length. Computing efficiency, however, needs not be a key comparison criterion between these two methods because both are already very efficient when compared to alignment-based methods. Most importantly, we showed empirically and theoretically that the TUP method outperforms the FFP-3 method. In addition, the FFP method does not use the information about the starting of the reading frame, which is usually known. We also provided a numerical measure for comparing various trees constructed.

As pointed out by a reviewer, the dataset under study contains only prokaryotic genomes, which have much simpler structures compared to eukaryotic genomes. Because eukaryotic genomes are complicated by their introns and exons, the proposed method might not be suitable for eukaryotic genomes.

For a better classification result with FFP-*k*, a much larger value of *k* than 3 was recommended in [4] but with the tradeoff of the much larger number of possible categories, i.e., 4^*k*^. For example, the number of possible categories is 4096 for *k*=6 or 262144 for *k*=9. The FFP-6 or FFP-9 method is expected to provide a better classifier than the classifier based on the FFP-3 method. For a fair comparison, method FFP-6 or FFP-9 should be compared to its TUP counterpart, the “extended TUP” method (say, TUP-6 or TUP-9), that uses multiple consecutive trinucleotides of the same length. The “extended TUP” method could be useful when the number of species to be classified is huge. Based on the entropy theory provided in this paper, we expect that the classifier based on this multiple-TUP method would be superior to the classifier based on the corresponding FFP-*k* method.
